# Integrating LCM-Based Spatio-Temporal Transcriptomics Uncovers Conceptus and Endometrial Luminal Epithelium Communication that Coordinates the Conceptus Attachment in Pigs

**DOI:** 10.3390/ijms22031248

**Published:** 2021-01-27

**Authors:** Feiyu Wang, Shilei Zhao, Dadong Deng, Weiwei Wang, Xuewen Xu, Xiaolei Liu, Shuhong Zhao, Mei Yu

**Affiliations:** Key Lab of Agricultural Animal Genetics, Breeding and Reproduction of Ministry of Education, College of Animal Science and Technology, Huazhong Agricultural University, Wuhan 430070, China; feiyuw@outlook.com (F.W.); 15766387510@163.com (S.Z.); dengdadong123@sohu.com (D.D.); WeiweiWang@webmail.hzau.edu.cn (W.W.); xuewen_xu@mail.hzau.edu.cn (X.X.); xiaoleiliu@mail.hzau.edu.cn (X.L.); shzhao@mail.hzau.edu.cn (S.Z.)

**Keywords:** spatio-temporal transcriptomics, laser capture microdissection, endometrial luminal epithelium, conceptus attachment, pigs

## Abstract

Attachment of conceptus to the endometrial luminal epithelium (LE) is a critical event for early placentation in Eutheria. Since the attachment occurs at a particular site within the uterus, a coordinated communication between three spatially distinct compartments (conceptus and endometrial LE from two anatomical regions of the uterus to which conceptus attaches and does not attach) is essential but remains to be fully characterized. Using the laser capture microdissection (LCM) technique, we firstly developed an approach that can allow us to pair the pig conceptus sample with its nearby endometrial epithelium sample without losing the native spatial information. Then, a comprehensive spatio-temporal transcriptomic profile without losing the original conceptus-endometrium coordinates was constructed. The analysis shows that an apparent difference in transcriptional responses to the conceptus exists between the endometrial LE from the two anatomically distinct regions in the uterus. In addition, we identified the communication pathways that link the conceptus and endometrial LE and found that these pathways have important roles in conceptus attachment. Furthermore, a number of genes whose expression is spatially restricted in the two different anatomical regions within the uterus were characterized for the first time and two of them (*SULT2A1* and *MEP1B*) may cooperatively contribute to establish conceptus attachment in pigs. The results from our study have implications in understanding of conceptus/embryo attachment in pigs and other large polytocous species.

## 1. Introduction

Successful embryo implantation is a critical prerequisite for establishment of placenta and ultimately determines pregnancy success rates. Collectively, the process of implantation includes two important stages: (1) initial attachment, and (2) firm attachment or invasion of the endometrium depending on the species [1,2]. Although strategies used for the second stage differ from species to species, adhesion of conceptus (embryo and associated extraembryonic membranes) to endometrial luminal epithelium (LE) is an important event that is common across species [3].

Pigs have a protracted implantation period. Conceptuses freely float in the uterus before approximately day 13 of gestation (gestation length in pigs is 114 days) [4]. Attachment of conceptus to the endometrial luminal epithelium is initiated around day 13 of gestation [5]. After a series of morphogenetic and physiological events, the attachment becomes stable by establishing a firm trophoblast-endometrial epithelial bilayer and ends with the formation of a non-invasive epitheliochorial placenta with intact endometrial epithelium separating maternal blood and fetal membranes around days 25–30 [6]. Previous studies have identified a number of genes and signaling pathways related to conceptus attachment in pigs, such as estrogen-mediated pregnancy signaling pathways [7,8,9,10,11]. However, the mechanisms remain to be better understood. 

Conceptus must select a particular site within the uterus for initial attachment. In the vertical direction, the uterus is demarcated into two anatomical locations: mesometrial (M) and anti-mesometrial (AM) sides, and initial attachment of pig conceptus normally takes place at the mesometrial side [12,13]. Several genes (such as *VEGF*, *IFN*-γ, *TNF-α*, *HIF-1α* and *ABCA1*) were identified to be differentially expressed in endometrium between the two anatomical locations of the uterus during implantation in pigs [14,15]. Our recent study investigated genes and miRNAs expressed in endometrial tissues from the two anatomical locations and detected important genes and pathways that might regulate the mesometrial-biased attachment in pigs [16]. The results indicate that the molecular and physiological signatures in endometrium vary spatially within the uterus. It is worth noting that the conceptus–endometrium interface is complex and consists of heterogeneous cell types/compartments including trophoblast cells, endometrial luminal and glandular epithelial cells, stromal cells, endothelial cells and other cell types. During the attachment, conceptus produces a number of signals which directly act on endometrial epithelium or are transduced by endometrial epithelium to other compartments of the endometrium to induce endometrial receptivity [4,7,8,17]. Recent studies also revealed that genes expressed in endometrial luminal epithelium showed distinct patterns, and endometrial luminal epithelium is the main target of the conceptus signals in pigs [18,19]. Taken together, our hypothesis is that the molecular features between endometrial luminal epithelium adjacent to the conceptus (mesometrial side: M) and luminal epithelium on the opposite side of the conceptus (considered as anti-mesometrial side: AM) may be different and therefore a coordinated communication between conceptus and endometrial luminal epithelium at the two sides is of importance for successful conceptus attachment.

Spatiotemporal transcriptomics is a powerful strategy for identification of the communication pathways in tissue or organ in the natural setting [20,21,22]. Recently, Biase et al. [23] developed paired conceptus-endometrium analyses to determine the crosstalk at the level of an individual pregnancy in cattle. However, it is technically much more difficult to pair each conceptus with its nearby endometrial epithelium sample in large polytocous species, such as pigs. Laser capture microdissection (LCM) is a powerful technique that can capture specific single cell or tissue while maintaining the structure and spatial information [24,25,26]. In the present study, to provide new insight into understanding coordinated endometrial epithelium responses to the implanting conceptus, intact pig uterine samples were collected at two critical implantation stages (gestational days 12 (pre-attachment stage) and 15 (attachment stage)), respectively. Then, the LCM technique was used to dissect the endometrial luminal epithelium at the mesometrial side (LE-M), the conceptus adjacent to the LE-M and the endometrial luminal epithelium at the anti-mesometrial (LE-AM) side from the same conceptus–endometrium interface, respectively. Thereafter, spatially and temporally resolved transcriptomes were constructed and the communication pathways between conceptus and endometrial luminal epithelium from different anatomical locations of the uterus were examined. 

## 2. Results

### 2.1. Transcriptomic Profiles of Three Spatially Distinct Compartments from Conceptus–Endometrium Interface in Pigs 

**Figure 1:**
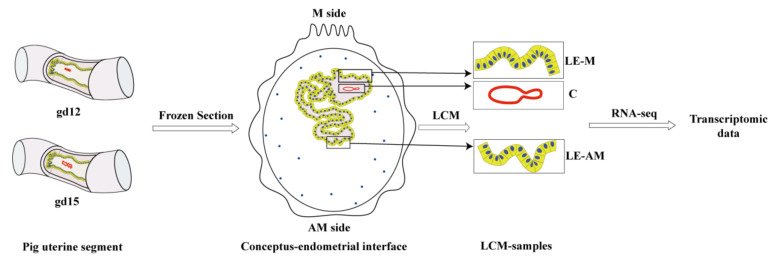
Schematic of the experimental workflow. Pig uterine segments in which conceptuses were not flushed out were collected from gestational days 12 (gd12, pre-attachment stage) and 15 (gd15, attachment stage) respectively, and were cross-sectioned by using a cryostat. Three spatially distinct compartments of the conceptus–endometrial interface from each stage were laser capture micro-dissected (LCM) and analyzed by RNA-seq to generate the spatial-temporal transcriptomic data. LE-M: endometrial luminal epithelium at the mesometrial side; LE-AM: endometrial luminal epithelium at the anti-mesometrial side; C: conceptuses.

Three spatially distinct compartments in the same uterine cross-section were laser capture micro-dissected (Figure 1). The three compartments include (1) endometrial luminal epithelium at the mesometrial side (named as LE-M) to which conceptus attaches, (2) conceptus adjacent to LE-M and (3) endometrial luminal epithelium at the anti-mesometrial side (named as LE-AM) to which conceptus does not attach. Therefore, six types of samples (3 compartments per gestational day) were used for RNA-sequencing and a total of 15,062 genes were detected. Genes that have been reported to be expressed in pig endometrial luminal epithelium (such as *SPP1* (Secreted phosphoprotein 1) [27,28], *MUC1* (Mucin 1) [29] and *STC1* (Stanniocalcin 1) [30]) were included, and pig conceptus-expressed genes including *IFNG* (Interferon gamma) [31], *PLET1* (Placenta expressed transcript 1) [32], *PLAC1* (Placenta-specific 1) [33] and *PLAC8* (Placenta-specific 8) [34,35] were also detected. Descriptive statistics of the RNA-seq data are given in Table S1.

### 2.2. Temporal Transcriptional Changes in the Three Spatially Distinct Compartments Before and at Attachment Stage 

A total of 1699 and 1607 genes were detected to be differentially expressed in LE-M and LE-AM obtained between before and at the attachment stage, respectively (Figures S1 and S2; Table S2). Most of the differentially expressed genes (DEGs) were greatly enriched in terms of extracellular exosome. Of the DEGs identified in the LE-M, genes related to the integrin-mediated signaling pathway were upregulated at the attachment stage. Of the DEGs identified in the LE-AM, genes involved in carbonate dehydratase activity were upregulated, but those genes related to cell surface were downregulated at the attachment stage (Table S3). Furthermore, we identified 989 DEGs in conceptus before and at the attachment stage. The most significantly enriched GO terms were extracelluar exosome and perinuclear region of cytoplasm (Figure S3; Tables S2 and S3). Therefore, the transcriptional regulation in the three spatially distinct compartments apparently differs between before and at the attachment stage.

### 2.3. Spatial Transcriptional Changes in LE between the Two Anatomical Locations within Uterus

**Figure 2:**
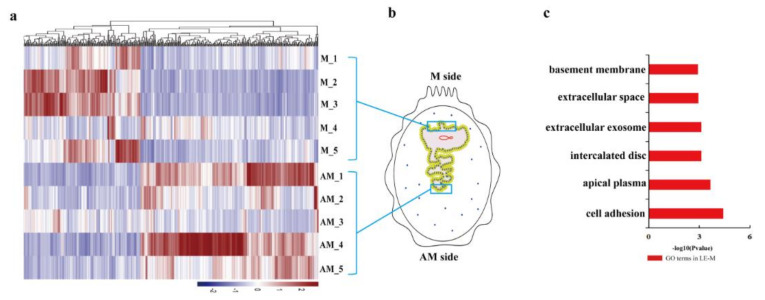
Heatmap and GO analysis for DEGs identified between LE-M and LE-AM on gd12 in pigs. (**a**) Heatmap for DEGs identified in LE-M compared to LE-AM on gd12. (**b**) Schematic illustration of the whole pig uterine cross-section taken from gestational day 12. (**c**) GO analysis of the upregulated DEGs identified in LE-M compared to LE-AM on gd12. DEGs: differentially expressed genes; LE-M: endometrial luminal epithelium at the mesometrial side; LE-AM: endometrial luminal epithelium at the anti-mesometrial side; gd12: gestational day 12 (pre-attachment stage).

**Figure 3:**
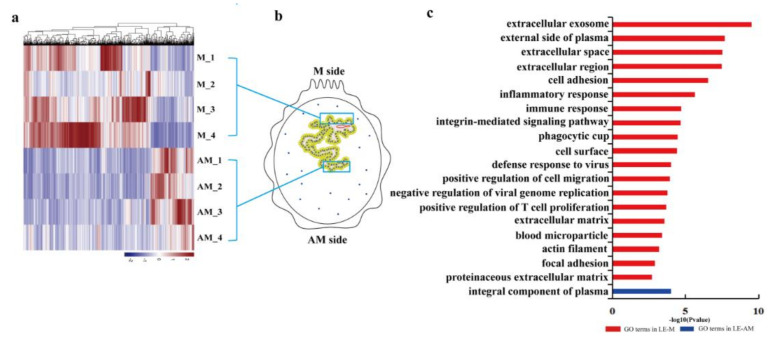
Heatmap and GO analysis for DEGs identified between LE-M and LE-AM on gd15 in pigs. (**a**) Heatmap for DEGs identified in LE-M compared to LE-AM on gd15. (**b**) Schematic illustration of the whole pig uterine cross-section taken from gestational day 15. (**c**) GO analysis of the upregulated DEGs identified in LE-M compared to LE-AM (red), and identified in LE-AM compared to LE-M (blue) on gd15. DEGs: differentially expressed genes; LE-M: endometrial luminal epithelium at the mesometrial side; LE-AM: endometrial luminal epithelium at the anti-mesometrial side; gd15: gestational day 15 (attachment stage).

Transcriptional changes between the endometrial luminal epithelium from the mesometrial side (LE-M) to which conceptus attaches and the anti-mesometrial side (LE-AM) to which conceptus does not attach were investigated. In total, 941 spatially differentially expressed genes (DEGs) were identified, in which 345 DEGs were detected in the pre-attachment stage, and 596 DEGs were detected at the attachment stage (Table S4). Most of the upregulated genes in LE-M at the two stages were mainly enriched in extracellular exosome (Figure 2 and Figure 3; Table S5). In addition, the majority of upregulated genes in the LE-M from before and at the attachment stage were significantly enriched in apical plasma membrane and inflammatory response respectively (Figure 2 and Figure 3; Table S5), whereas terms specific for the upregulated genes in the LE-AM at the attachment stage were mainly related to the integral component of the plasma membrane (Figure 2 and Figure 3; Table S5). Thus, the findings showed that at the attachment stage, molecular signatures of the endometrial luminal epithelium vary greatly by anatomical location within the uterus. 

### 2.4. Communication Pathways Linking the Conceptus and Endometrial Luminal Epithelium at the Attachment Stage

The analyses revealed the signaling pathways in which the DEGs from both conceptus and endometrial luminal epithelium participated (Table S6). Most of these pathways are related to conceptus attachment, including extracellular space, SPP1-mediated cell adhesion pathways (such as focal adhesion and integrin-mediated signaling pathway) and estrogen metabolism and signaling pathways (such as metabolic pathways and the PI3K-Akt signaling pathway) (Table S6). 

### 2.5. Associations between Genes with Spatially Restricted Expression Patterns in LE within Uterus and Conceptus Attachment

**Figure 4:**
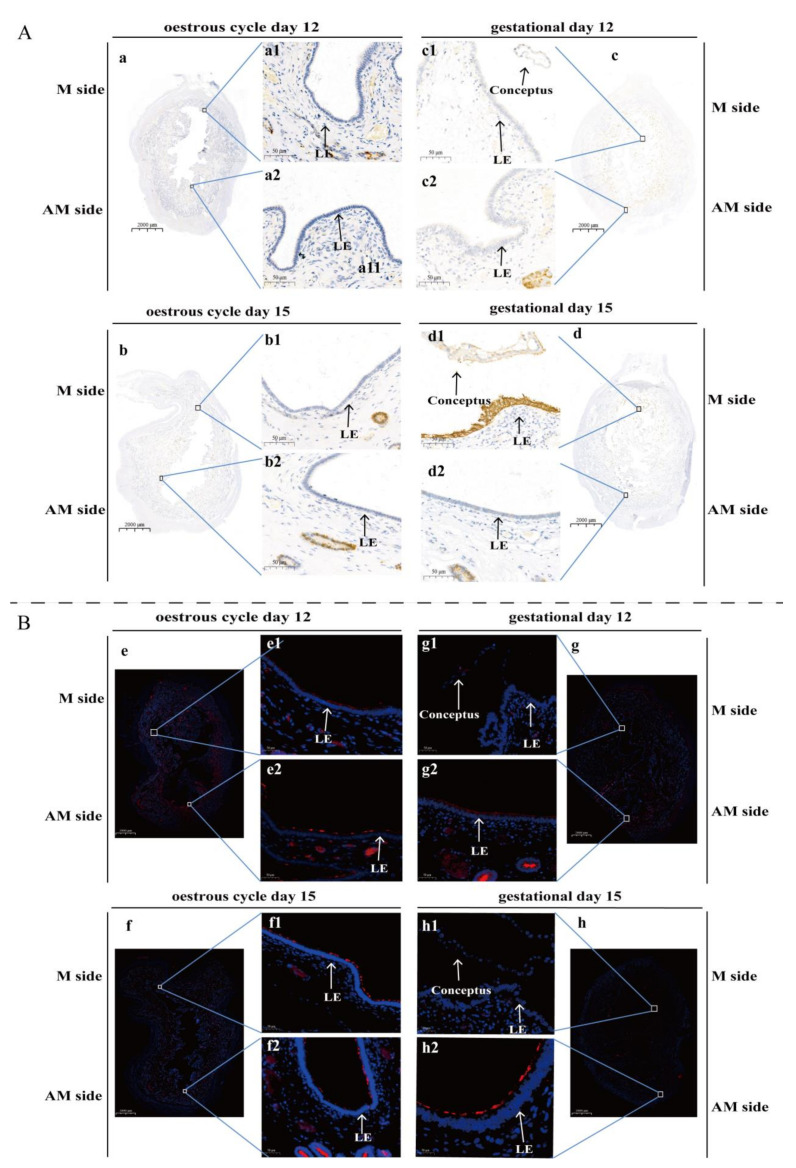
Spatial expression pattern of SULT2A1 and MEP1B in endometrial luminal epithelium within uterus of pigs. (**A**) Immunohistochemical analysis of SULT2A1 in the whole uterine cross-section of pigs (*n* = 3 gilts/estrous cycle day or gestational day). (**B**) Immunofluorescence analysis of MEP1B in the whole uterine cross-section of pigs (*n* = 3 gilts/estrous cycle day or gestational day). Representative images taken from the whole uterine cross-section at estrous cycle day 12 (**a**,**e**), estrous cycle day 15 (**b**,**f**), gestational day 12 (**c**,**g**) and gestational day 15 (**d**,**h**), respectively. Higher magnification images are shown of the localization of SULT2A1 in endometrial regions from the M side and AM side of the uterus respectively, and were taken from the same uterine cross-section at estrous cycle day 12 (**a1**,**a2**), estrous cycle day 15 (**b1**,**b2**), gestational day 12 (**c1**,**c2**) and gestational day 15 (**d1**,**d2**), respectively. Higher magnification images are shown of the localization of MEP1B in endometrial regions from the M side and AM side of the uterus respectively, and were taken from the same uterine cross-section at estrous cycle day 12 (**e1**,**e2**), estrous cycle day 15 (**f1**,**f2**), gestational day 12 (**g1**,**g2**) and gestational day 15 (**h1**,**h2**), respectively. LE: luminal epithelium; M side: mesometrial side; AM side: anti-mesometrial side. Scale bars = 50 µm.

**Figure 5:**
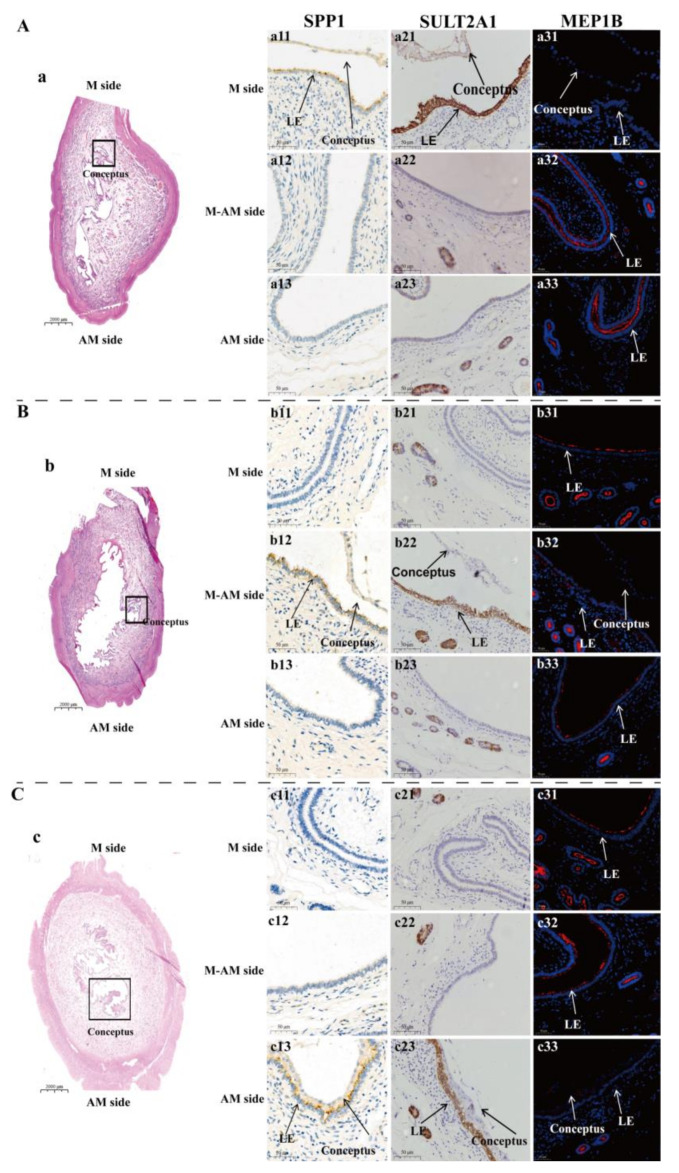
Expression of SPP1, SULT2A1 and MEP1B in the endometrial luminal epithelium in two types of pig uterine samples defined in terms of conceptus localization in the uterus at gestational day 15. (**A**) Representative images taken from uterine cross-section in which conceptus is located in the mesometrial side (*n* = 3 gilts). (**B**,**C**) Representative images taken from uterine cross-section in which conceptus is located away from the mesometrial side (*n* = 3 gilts, including uterine cross-sections in which conceptus is located between mesometrial side and anti-mesometrial side and in anti-mesometrial side, respectively.) (**a**–**c**) Hematoxylin and eosin-stained whole uterine cross-section. Higher magnification images were taken from the same uterine cross-section and show the localization of the 3 proteins in endometrial regions from the M side ((**a11**,**b11**,**c11**) for SPP1; (**a21**,**b21**,**c21**) for SULT2A1; (**a31**,**b31**,**c31**) for MEP1B), between M and AM side ((**a12**,**b12**,**c12**) for SPP1; (**a22**,**b22**,**c22**) for SULT2A1; (**a32**,**b32**,**c32**) for MEP1B) and AM side ((**a13**,**b13**,**c13**) for SPP1; (**a23**,**b23**,**c23**) for SULT2A1; (**a33**,**b33**,**c33**) for MEP1B) of the uterus, respectively. LE: luminal epithelium; M side: mesometrial side; AM side: anti-mesometrial side. Scale bars = 50 µm.

Of the DEGs identified between the LE-M and LE-AM at the attachment stage, *SULT2A1* (Sulfotransferase family 2A member 1) and *MEP1B* (Meprin A subunit beta) are two of the most significantly differentially expressed genes. *SULT2A1* was significantly highly expressed in the LE-M; in contrast, *MEP1B* was significantly highly expressed in the LE-AM (Figure S4; Table S4). Furthermore, immunohistochemical and immunofluorescence assays were performed on the whole pig uterine cross-sectional samples from: (1) days 12 and 15 of the estrous cycle, and (2) days 12 and 15 of gestation. On days 12 and 15 of the estrous cycle, the endometrial luminal epithelium was completely negative for SULT2A1 but contained scattered positive signals for MEP1B. Similarly, on day 12 of gestation (pre-attachment stage), the endometrial luminal epithelium remained negative for SULT2A1 and showed scattered positive signals for MEP1B (Figure 4 and Figures S5–S12). However, on day 15 of gestation (attachment stage), SULT2A1 was abundantly detected in the LE-M but completely absent in the LE-AM; in contrast, MEP1B was completely absent in the LE-M but greatly expressed in the LE-AM (Figure 5). 

SPP1 is an adhesive molecule that can mediate adhesion of conceptus to the endometrial luminal epithelium [27,28,36,37,38,39]. We then examined the expression pattern of SPP1 in the uterine cross-sections from two types of samples defined in terms of conceptus localization in the uterus: conceptus is located at the mesometrial side or away from the mesometrial side (Figure 5). The results showed that wherever the conceptus is located, SPP1 was observed only in the endometrial luminal epithelium in close proximity to conceptus (Figure 5). The findings indicate that adhesion of conceptus can occur in different regions of the uterus in pigs. Furthermore, the expression pattern of SULT2A1 and MEP1B was investigated on the two types of samples, respectively. SULT2A1 showed exactly the same pattern as that of SPP1 in that wherever the conceptus is located, SULT2A1 was abundantly presented in the endometrial luminal epithelium in close proximity to conceptus but completely absent in the endometrial luminal epithelium located away from conceptus. In contrast to SULT2A1 and SPP1, MEP1B was completely absent in the endometrial luminal epithelium in close proximity to conceptus but greatly expressed in the endometrial luminal epithelium located away from conceptus. Thus, SULT2A1 and MEP1B were greatly expressed in two different anatomical uterine regions to which conceptus attaches and does not attach, respectively. 

## 3. Discussion

Synchronous communication between conceptus and the endometrial luminal epithelium surrounding the conceptus is of importance for conceptus attachment. Coupling LCM and RNA-seq techniques, we captured the conceptus and its surrounding endometrial luminal epithelium from the intact conceptus–endometrium interface at two critical implantation stages of pigs and constructed spatio-temporal transcriptomic profiles of the conceptus–endometrial luminal epithelium interface without losing the native spatial information. Then, we identified the communication pathways linking the conceptus and endometrial luminal epithelium and found that these pathways have important roles in conceptus attachment. We also characterized that genes with spatial-restricted expression patterns in the endometrial luminal epithelium may cooperate to establish the attachment of conceptus to the endometrial luminal epithelium.

The pig is a polytocous species, and it is technically difficult to establish a spatially one-to-one correspondence between a conceptus and its surrounding uterine tissue. In the present study, the intact whole uterus in which the conceptuses were not flushed out were used for cryosection. Using the LCM technique, three spatially distinct compartments (LE-M, conceptus and LE-AM) were captured from the same conceptus–endometrial interface. As expected, those genes that have been demonstrated to be expressed in porcine endometrial epithelium and conceptus were included in our datasets, respectively. In addition, some of the genes we detected are different from those reported by Zeng et al. [18,19]. The difference could be attributed to (1) the differences in pig breeds and sample collection strategies used in the two studies, and (2) the difference in samples captured since the endometrial luminal epithelium was captured separately from two anatomical regions in the uterus in the present study. Taken together, our results indicate the usability of the approach we developed in investigating the communication between conceptus and its surrounding endometrial luminal epithelium without losing the original conceptus–endometrium coordinates in pigs and other polytocous species.

A large number of DEGs were identified between pre-attachment stage and attachment stage, indicating that the expression patterns in porcine endometrial luminal epithelium differ greatly before and after conceptus attachment. In addition, of the DEGs that were identified between LE-M and LE-AM, the number of DEGs detected at the attachment stage is higher than those DEGs detected at the pre-attachment stage. The findings provided further information to show that a different response to conceptus exists between LE-M and LE-AM, and the difference became greater when conceptus attaches to the endometrial luminal epithelium [14,15,16]. Exosomes carry proteins, lipid and nucleic acids and participate in maternal–embryonic interaction [40,41]. Consistently, majority of the DEGs were greatly overrepresented in functional term of extracellular exosome. The genes include those that have been characterized to be involved in conceptus attachment, such as *SPP1* and *ITGAV* [4,11,27,42]. SPP1 is an adhesive molecule that mediates porcine endometrial epithelium–conceptus adhesion through interaction with multiple integrin subunits, including ITGAV [43]. The findings indicate that exosomes play important roles in the conceptus–endometrial epithelium interaction during conceptus attachment in pigs, which is consistent with previous reports [18,42,44,45,46,47].

Interleukin 1 beta 2 (IL1B2), released specifically by pig conceptus at its elongation time (at gestational day 12, pre-attachment stage), is an alternative transcript of pro-inflammatory IL1B [48,49]. IL1B2 can mediate conceptus elongation and active nuclear factor kappa-B (NF-κB) by upregulating NF-κB responsive genes (*PTGS2* and *IKBA*) in the endometrial luminal epithelium [49,50]. Consistently, *IL1B2* was only expressed in the conceptuses micro-dissected from gestational day 12 and expressions of a number of genes in the nuclear factor kappa-B (NF-κB) pathway were higher in the LE-M on gestational day 12. These genes include those encoding (1) interleukin 1 receptor type 1 (IL1R1) and interleukin 1 receptor accessory protein (IL1RAP), (2) three NF-κB subunits (RELB, REL and NFKB2/p52) and (3) three members of the NF-kappa-B inhibitor (I-kappa-B) family. We further found that the matrix metalloproteinase-8 (MMP8) gene was rapidly and transiently induced in the endometrial luminal epithelium on gestational day 12 but undetectable on gestational day 15, indicating that an increased expression of *MMP8* in the endometrial luminal epithelium is temporally associated with the increased expression of *IL1B2* in the conceptus (Table S2). Several studies showed that IL1B can promote the secretion of metalloproteinases via activation of transcription factors, such as CCAAT/enhancer binding protein beta (CEBPB) [51,52]. Consistently, we found that CEBPB mRNA was also upregulated in the endometrial luminal epithelium on gestational day 12. In addition, MMP8 belongs to the fibrillar collagenase family and plays roles in inflammation and tissue remodeling [53]. Thus, our findings suggest that, in addition to mediating the NF-κB pathway, the increased expression of *IL1B2* in pig conceptus may also induce *MMP8* expression for endometrial luminal epithelium remodeling.

SULT2A1 is a member of the hydroxysteroid sulfotransferase subfamily and able to catalyze the sulfation of steroids, including 17β-estradiol (E2), to the sulfate forms [54,55,56]. The sulfoconjugates are inactive forms of steroids and can be transported to target tissues or cells to become biologically active via the sulfatase pathway [57,58,59]. Previous studies have shown that estrogen sulfates were increased in the uterine fluid during implantation of pigs [60,61]. This led us to assume that the increased expression of *SULT2A1* in the endometrial luminal epithelium may play important roles in regulation of steroidogenesis and estrogen homeostasis in conceptus and the endometrial luminal epithelium. MEP1B is a membrane-bound metalloprotease which is involved in cleavage of several pro-inflammatory cytokines and extracellular matrix proteins and thus plays roles in regulating inflammation and tissue remodeling [62,63,64]. A previous study also found that MEP1B knockout mice have decreased litter sizes compared to wild-type litters [65]. The communication pathway analysis revealed that SPP1-mediated integrin pathways are related to conceptus–endometrial epithelium adhesion. In agreement with the previous reports, we found that *SPP1* and integrin subunits were upregulated on gestational day 15 in endometrial epithelium and conceptus, respectively. Pig conceptus-derived estrogens (mainly E2) are the key factors for establishment of pregnancy. The estrogens are secreted at two periods during implantation: (1) an increase in secretion between days 11 and 12 of gestation, and (2) a sustained increase in secretion from day 15 to 25 of gestation to initial attachment of conceptus to the endometrial luminal epithelium [66,67,68]. Previous studies showed that E2 can induce the expression of *SULT2A1* and *SPP1* but has an effect to downregulate the expression of *MEP1B,* and the effect of the estrogens is restricted to the region in close proximity to conceptus [10,35,69,70,71]. These results may explain the spatial-restricted expression manner of SULT2A1 and SPP1 in the uterus. In addition, the findings that MEP1B and SPP1 exhibited spatially specific mutually exclusive patterns of expression within the uterus at the attachment stage could be explained by the previous reports that SPP1 can be effectively degraded by MEP1B [62,72]. Taken together, the findings led us to hypothesize a possible model for conceptus attachment: wherever the conceptus comes into apposition, it produces estrogens to act on the endometrial luminal epithelium juxtaposed to itself and induce steroidogenesis-related genes, such as *SULT2A1*, to regulate estrogen homeostasis. At the same time, *SPP1* is induced to mediate endometrial epithelium–conceptus adhesion, and importantly, *MEP1B* is simultaneously suppressed in the same region to maintain the adhesion.

In conclusion, we constructed a comprehensive spatio-temporal transcriptomic dataset of the conceptus–endometrial luminal epithelium interface during two critical conceptus attachment stages in pigs and detected novel pathways related to conceptus attachment. Transcriptional profiles that correspond to two anatomically distinct regions in the uterus at each stage were identified and the coordinated endometrial epithelium responses to the conceptus were uncovered.

## 4. Materials and Methods

### 4.1. Sample Collection

All animal procedures were performed according to protocols approved by the Ethics Committee of Huazhong Agricultural University (HZAUSW-2016-015, 5 January 2016). Yorkshire gilts were checked for estrus twice daily and bred at the onset of the second estrus (Day 0) and again 12 h later. Uterine samples were collected on gestational days 12 (gd12, *n* = 7) and 15 (gd15, *n* = 8), respectively. The uterus was cut into 12–15 cm segments with sterile scalpel blades and numbered in sequence. Several uterine segments were randomly selected from each gilt and flushed with cold RNase-free phosphate buffer saline. If the pregnancy was confirmed by the presence of conceptuses in uterine flushings, the uterine samples without being flushed were fixed immediately in 10% neutral-buffered formalin for 24 h followed by paraffin embedding (FFPE) or embedded in precooled optimum cutting temperature (OCT) compound (SAKURA Tissue-Tek O.C.T. Compound 4583, Torrance, CA, USA), snap-frozen in liquid nitrogen and stored at −80 °C. In addition, uterine samples on days 12 (*n* = 3) and 15 (*n* = 3) of the estrus cycle were collected without being flushed and further processed as described above. 

### 4.2. Laser Capture Microdissection and RNA Library Construction 

Two frozen uterine segments from each gilt (4 and 5 gilts from gestational days 12 and 15, respectively) were randomly selected for laser capture microdissection. Each segment was cross-sectioned to 13 μm-thick slices using a cryostat (Leica CM1950, Leica Biosystems, Heidelberger, Germany). The serial sections were collected and mounted onto membrane slides (MembraneSlide Nuclease and Human nucleic acid-free PEN-Membrane 2.0 um, No.11505189, Leica). The slides were processed through a graded series of ethanol for fixation and dehydration according to a protocol reported by Chen et al. [73]. All solutions were prepared with RNase-free water. After the slides were air-dried, the laser capture microdissection was performed immediately by using a Leica LMD 7000 laser microdissection system (Leica, Frankfurt, Germany) according to the instructions. Only those sections that show the presence of conceptus were used for laser capture microdissection. In each section from each segment, the targets we captured were the endometrial luminal epithelium cells at the attachment site (mesometrial side, named as LE-M), endometrial luminal epithelium cells at anti-mesometrial to the attachment site (named as LE-AM) and conceptus. The LE-M, LE-AM or conceptus captured from the same segment were pooled. Total RNA was isolated immediately using the miRNeasy Micro Kit (QIAGEN, No. 217084, Düsseldorf, Germany) following the manufacturer’s instructions. 

Prior to library preparation, integrity of RNA was checked with an Agilent Bioanalyzer 2100 (Agilent Technologies, Santa Clara, CA, USA) and concentrations of RNA were determined using a NanoDrop spectrophotometer (Thermo Scientific, Waltham, MA, USA). In total, libraries were constructed using RNA yielded from each target captured from different gestational days, including 5 libraries for LE-M on gestational day 12 (1–2 segments/gilt, 3 gilts, named as gd12-LE-M), 4 libraries for LE-M on gestational day 15 (1 segment/gilt, 4 gilts, named as gd15-LE-M), 5 libraries for LE-AM on gestational day 12 (1–2 segments/gilt, 3 gilts, named as gd12-LE-AM), 4 libraries for LE-AM on gestational day 15 (1 segment/gilt, 4 gilts, named as gd15-LE-AM), 4 libraries for conceptus on gestational day 12 (1 segment/gilt, 4 gilts, named as gd12-C) and 4 libraries for conceptus on gestational day 15 (1–2 segments/gilt, 3 gilts, named as gd15-C).

### 4.3. RNA-Sequencing 

All libraries were sequenced on the Illumina HiSeq X Ten system with a paired-end read length of 2 × 150 bp. The obtained clean data (Fastq files) were quality controlled with the fastaQC (version 0.11.7) and 10 bp was removed from the start of the reads with the fastp (version 0.19.5) [74]. Sequences were mapped to the *Sus scrofa* genome assembly 11.1 (ENSEMBLE: ftp://ftp.ensembl.org/pub/release-96/gtf) with Hisat2 [75] and then the duplicates were removed with samtools (version 1.9) [76]. Reads counts for each gene from each library was calculated using HTSeq [77]. A detailed sample description is given in Table S1. Differentially expressed genes (DEGs) were determined using DESeq2 [78]. In this study, two comparisons (LE-M vs. LE-AM on gd12 and LE-M vs. LE-AM on gd15, respectively) were performed using the paired sample test to detect the transcriptional changes in the endometrial epithelium that may arise from differences in anatomical location of the uterus. The other three comparisons (LE-M from gd12 vs. LE-M from gd15, LE-AM from gd12 vs. LE-AM from gd15 and conceptuses from gd12 vs. those from gd15, respectively) were carried out using the independent sample test to detect the transcriptional changes that may arise from differences in developmental stage. The genes with adjusted *p*-value < 0.05 and |fold-change| > 1 were considered DEGs. Gene Ontology (GO) analysis for the DEGs was carried out using DAVID [79]. In addition, to investigate the communication pathways using the spatio-temporal transcriptomic dataset in which the spatial information is maintained, we combined the DEGs identified in conceptus and LE-M between stages with those DEGs detected between LE-M and LE-AM at the attachment stage. Then, the Gene Ontology (GO) analysis was performed on the combined dataset. In this study, GO terms with adjusted *p*-value < 0.05 were considered significantly enriched. 

### 4.4. Quantitative Real-Time PCR

Total RNA was reverse transcribed using the PrimeScript RT Reagent Kit with gDNA Eraser (Takara Biomedical Technology, Dalian, China). The qRT-PCR was performed using standard SYBR Premix Ex Taq II (Tli RNaseH Plus) (Takara Biomedical Technology, Dalian, China) in a Bio-Rad CFX96 Touch Real-Time PCR Detection System (Bio-Rad Laboratories, Inc., Hercules, CA, USA). PCR conditions were as follows: a single cycle of 30 s at 95 °C, followed by 39 cycles of 5 s at 95 °C and 30 s at 60 °C. The gene-specific primers are listed in Table S7. The *GAPDH* gene was used as a control [80]. Wilcoxon’s test was performed using R, and *p*-values < 0.05 were considered statistically significant.

### 4.5. Histologic, Immunohistochemical and Immunofluorescence Analyses

The paraffin-embedded uterine samples were cross-sectioned into 4 μm-thick slices. After deparaffinization and rehydration, the sections were stained with hematoxylin and eosin for further histologic analysis or subjected to immunohistochemical and immunofluorescence analyses.

Immunohistochemical and immunofluorescence assays were performed as previously described [15]. Briefly, the uterine cross-sections were treated with 3% hydrogen peroxide (H_2_O_2_) to block the endogenous peroxidase activity and then subjected to heat-induced epitope retrieval and blocked with 5% bovine serum albumin (BSA) in phosphate-buffered saline (PBS) for 30 min at room temperature. In immunohistochemical assays, sections were incubated with SULT2A1 antibody (1:100, ab194113, Abcam, Cambridge, UK) and SPP1 antibody (1:100, D121078, Sangon Biotech, Shanghai, China) at 4 °C overnight and then incubated with secondary antibody (Goat anti-rabbit IgG-Biotin, SA1022, Wuhan Boster Biological Technology, Ltd., Wuhan, China) for 30 min. Slides were developed with diaminobenzidine (DAB) and counterstained with hematoxylin and mounted using coverslips. In immunofluorescence assays, the primary antibodies used were MEP1B polyclonal antibody (1:100, CSB-PA618098ESR1HU, CUSABIO, Wuhan, China). The corresponding secondary antibodies were Cy3 conjugated Goat Anti-rabbit IgG (H + L) (red) (1:100, GB21303, Servicebio, Wuhan, China) and counterstained with DAPI (blue). For each sample, a negative control (NC) was performed by replacing the primary antibody with PBS. 

All slides were scanned using the 3D HISTECH Panoramic Midi Scanner (3D HISTECH Ltd., Budapest, Hungary). The whole uterine cross-sectional images were taken and then subjected to analysis using CaseViewer software (3D HISTECH Ltd., Budapest, Hungary).

## Data Availability

The RNA-seq data generated were deposited into the NCBI (national center for biotechnology information) Sequence Read Archive database (PRJNA668716).

## Supplementary Materials

The following are available online at https://www.mdpi.com/1422-0067/22/3/1248/s1.
