# Supplementary material for: Integrating LCM-Based Spatio-Temporal Transcriptomics Uncovers Conceptus and Endometrial Luminal Epithelium Communication that Coordinates the Conceptus Attachment in Pigs
Source: Int J Mol Sci. 2021 Jan 27;22(3):1248. doi: 10.3390/ijms22031248 (PMC7866100; doi:10.3390/ijms22031248)
Supplement: Supplementary file 1 [file ijms-22-01248-s001.zip › Supplementary files/Supplement Figures.doc]

**Figure S1.** Heatmap and GO analysis for DEGs identified in LE-M between gd12 and gd15. (a) Heatmap for DEGs identified in LE-M between gd12 and gd15; (b) GO analysis of the up-regulated DEGs identified in LE-M on gd15. DEGs: differentially expressed genes; LE-M: endometrial luminal epithelium at the mesometrial side; gd12: gestational day 12 (pre-attachment stage); gd15: gestational day 15 (attachment stage).

**Figure S2. Heatmap and Gene Ontology (GO) analysis for DEGs identified in LE-AM between gd12 and gd15.** (a) Heatmap for DEGs identified in LE-AM between gd12 and gd15; (b) Gene Ontology (GO) analysis of the up-regulated DEGs identified in LE-AM on gd15 (red) and gd12 (blue). DEGs: differentially expressed genes; LE-AM: endometrial luminal epithelium at the anti-mesometrial side; gd12: gestational day 12 (pre-attachment stage); gd15: gestational day 15 (attachment stage) .

**Figure S3.** Heatmap and GO analysis for DEGs identified in C between gd12 and gd15. (a) Heatmap for DEGs identified in C between gd12 and gd15; (b) GO analysis of the up-regulated DEGs identified in C on gd15 (red) and gd12 (blue). DEGs: differentially expressed genes; C: conceptus; gd12: gestational day 12 (pre-attachment stage); gd15: gestational day 15 (attachment stage).

**Figure S4.** The qRT-PCR validation of *SULT2A1* and *MEP1B*. Data are represented as mean ± SEM, n = 3 or 4 gilts. **P* < 0.05, ***P* < 0.01. gd15-LE-M: endometrial luminal epithelium at the mesometrial side on gestational day 15; gd15-LE-AM: endometrial luminal epithelium at the anti-mesometrial side on gestational day 15.

**Figure S5.** Spatial expression pattern of SULT2A1 in the endometrial luminal epithelium within uterus of pigs at oestrous cycle day 12. Representative image of the entire cross section (left). Screenshots captured sequentially from the zoomed in image (right).

**Figure S6.** Spatial expression pattern of SULT2A1 in the endometrial luminal epithelium within uterus of pigs at oestrous cycle day 15. Representative image of the entire cross section (left). Screenshots captured sequentially from the zoomed in image (right).

**Figure S7.** Spatial expression pattern of SULT2A1 in the endometrial luminal epithelium within uterus of pigs at gestational day 12. Representative image of the entire cross section (left). Screenshots captured sequentially from the zoomed in image (right).

**Figure S8.** Spatial expression pattern of SULT2A1 in the endometrial luminal epithelium within uterus of pigs at gestational day 15. Representative image of the entire cross section (left). Screenshots captured sequentially from the zoomed in image (right).

**Figure S9.** Spatial expression pattern of MEP1B in the endometrial luminal epithelium within uterus of pigs at oestrous cycle day 12. Representative image of the entire cross section (left). Screenshots captured sequentially from the zoomed in image (right).

**Figure S10.** Spatial expression pattern of MEP1B in the endometrial luminal epithelium within uterus of pigs at oestrous cycle day 15. Representative image of the entire cross section (left). Screenshots captured sequentially from the zoomed in image (right).

**Figure S11.** Spatial expression pattern of MEP1B in the endometrial luminal epithelium within uterus of pigs at gestational day 12. Representative image of the entire cross section (left). Screenshots captured sequentially from the zoomed in image (right).

**Figure S12.** Spatial expression pattern of MEP1B in the endometrial luminal epithelium within uterus of pigs at gestational day 15. Representative image of the entire cross section (left). Screenshots captured sequentially from the zoomed in image (right).
