# Supplementary material for: Integrating LCM-Based Spatio-Temporal Transcriptomics Uncovers Conceptus and Endometrial Luminal Epithelium Communication that Coordinates the Conceptus Attachment in Pigs
Source: Int J Mol Sci. 2021 Jan 27;22(3):1248. doi: 10.3390/ijms22031248 (PMC7866100; doi:10.3390/ijms22031248)
Supplement: Supplementary file 1 [file ijms-22-01248-s001.zip › Supplementary files/Table S7.doc]

**List of primers used for qRT-PCR**

| **Gene name** | **Primer Sequence (5'->3')** | **Product length** | **GeneBank Accession No.** |
| --- | --- | --- | --- |
| *MEP1B* | F: TGGTGGACTTGACCGGGATA | 176 | XM_003127883.5 |
|  | R: CTCTGGCATTCATTTCCAAGC |  |  |
| *SULT2A1* | F: GAAGAGGAGGTGCGGTTTGA | 136 | NM_001037150.1 |
|  | R: TGGTTCCTGATTTGGGAAAAGTC |  |  |
| *GAPDH* | F: TATGATTCCACCCACGGCAAG | 138 | NM_001206359.1 |
|  | R: ATACGTAGCACCAGCATCACC |  |  |
